# Supplementary material for: A whole-animal phenotypic drug screen identifies suppressors of atherogenic lipoproteins
Source: eLife. 2026 Aug 27;14:RP105314. doi: 10.7554/eLife.105314 (PMC13521566; doi:10.7554/eLife.105314)
Supplement: Supplementary file 2 [file elife-105314-code1.zip › Output_data/JHDL_hit_summary_boxplot_1.pdf]

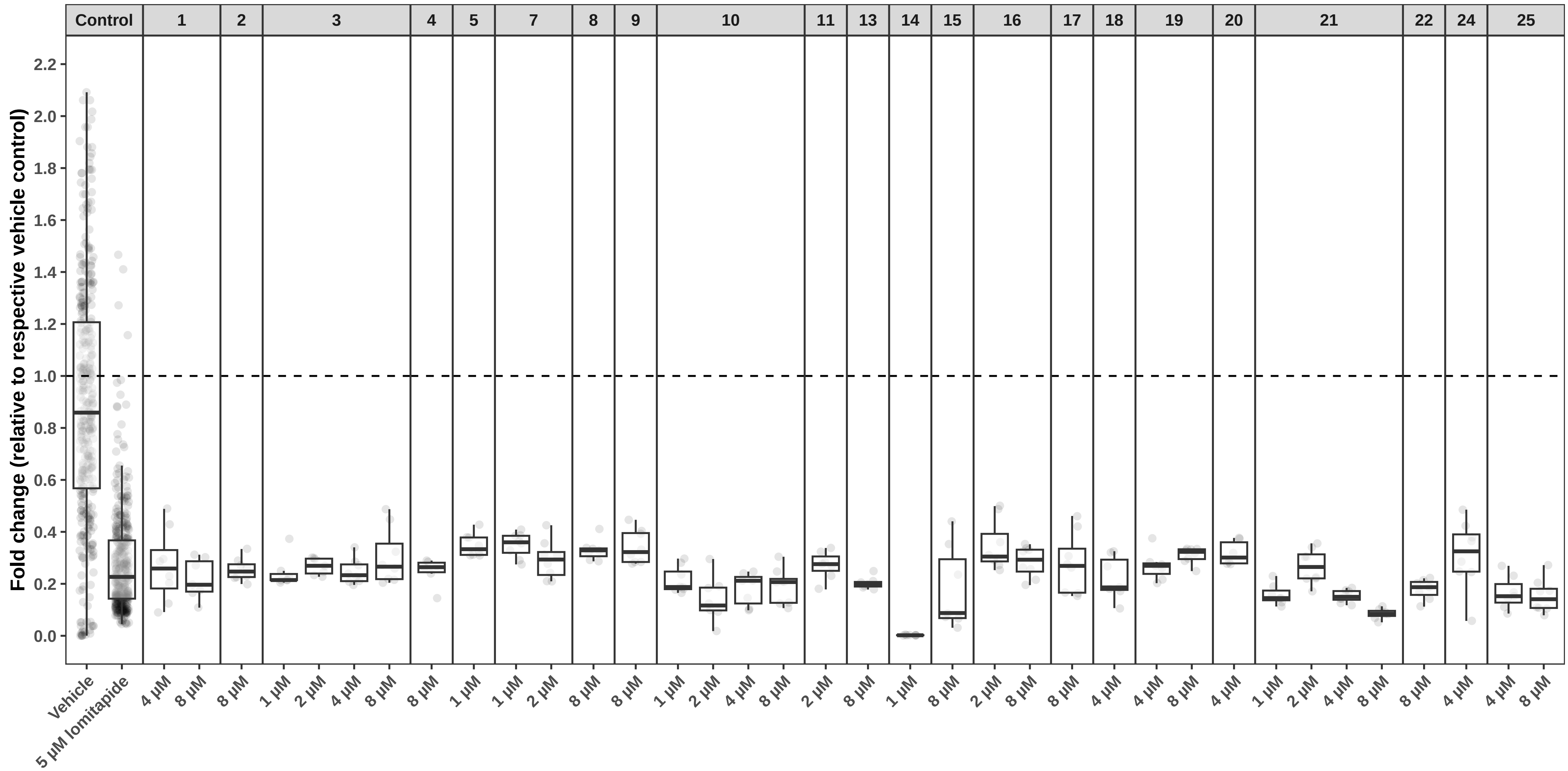

Control
1
2
3
4
5
7
8
9
10
11
13
14
15
16
17
18
19
20
21
22
24
25
Fold change (relative to respective vehicle control)
2.2
2.0
1.8
1.6
1.4
1.2
1.0
0.8
0.6
0.4
0.2
0.0
Vehicle
5 µM lomitapide
4 µM
8 µM
8 µM
1 µM
2 µM
4 µM
8 µM
8 µM
1 µM
1 µM
2 µM
8 µM
8 µM
1 µM
2 µM
4 µM
8 µM
2 µM
8 µM
1 µM
8 µM
2 µM
8 µM
8 µM
4 µM
4 µM
8 µM
4 µM
1 µM
2 µM
4 µM
8 µM
8 µM
4 µM
4 µM
8 µM
